# PES-Ag_3_PO_4_/g-C_3_N_4_ Mixed Matrix Film Photocatalyst for Degradation of Methyl Orange Dye

**DOI:** 10.3390/polym13111746

**Published:** 2021-05-27

**Authors:** Hayati Mohamad Mukhair, Abdul Halim Abdullah, Zulkarnain Zainal, Hong Ngee Lim

**Affiliations:** 1 Institute of Advanced Technology, Universiti Putra Malaysia, Serdang 43400, Selangor, Malaysia; hayatimukhair@gmail.com (H.M.M.); zulkar@upm.edu.my (Z.Z.); hongngee@upm.edu.my (H.N.L.); 2 Department of Chemistry, Faculty of Science, Universiti Putra Malaysia, Serdang 43400, Selangor, Malaysia

**Keywords:** film photocatalyst, PES-Ag_3_PO_4_/g-C_3_N_4_, visible light photocatalyst, wastewater treatment, methyl orange, Ag_3_PO_4_

## Abstract

In the present study, we explored the effectiveness of PES-Ag_3_PO_4_/g-C_3_N_4_ film photocatalyst in degrading methyl orange dye under visible light irradiation. The PES-Ag_3_PO_4_/g-C_3_N_4_ film photocatalyst was prepared via a non-solvent-induced phase inversion process and characterized by X-ray diffraction (XRD), scanning electron microscopy (SEM), laser scanning microscopy (LSM), X-ray photoelectron spectra (XPS), UV-diffuse reflectance (DRS), and water contact angle. The incorporation of the Ag_3_PO_4_/g-C_3_N_4_ composite into the PES matrix improved the pristine PES film’s hydrophilicity, as evidenced by the reduction of water contact angle from 79.03° to 54.33° for a film containing 15 wt % of Ag_3_PO_4_/g-C_3_N_4_ composite. The film’s photoactivity showed that 13 wt % was the best loading of Ag_3_PO_4_/g-C_3_N_4_ composite, and the degradation performance was maintained up to three cycles. The •O_2_^−^ and h^+^ were the predominant species responsible for the methyl orange degradation.

## 1. Introduction

Textile industries wastewater contains many types of dyes and chemical substances that pose an environmental challenge in wastewater disposal issues. Dye substances are toxic toward microorganisms, and their intense color blocks sunlight from penetrating the water, which causes severe problems for the aquatic ecosystem. Azo dyes, the largest group of synthetic colorants, are usually used in the textile industry. These dyes possess high stability against light, temperature, chemicals, and microbial attack, making conventional wastewater treatment less efficient. Moreover, the use of traditional physical and chemical methods produces secondary pollutants [1].

Advanced oxidation processes (AOPs) are the technologies that generally use hydroxyl radicals, the ultimate oxidant for remediating organic contaminants in wastewater by converting recalcitrant pollutants into biodegradable compounds. The efficacy of AOPs depends on the generation of reactive free radicals, mainly the hydroxyl radicals. The most favorable AOPs in wastewater treatment include Fenton oxidation [2], catalytic ozonation [3], and semiconductor-based heterogeneous photocatalysis.

In the past decades, heterogenous photocatalysis using transition metal oxides as catalysts stood out as a promising candidate in treatment methods because of its low cost and environmentally benign properties. Photocatalysis could mineralize organic compounds directly under solar irradiation and under mild conditions of temperature and pressure. However, the photocatalysts available to date are generally limited by either low photocatalytic efficiency in the visible light range or insufficient charge separation ability [4].

Recently, much effort is focused on the exploration of visible light active semiconductor photocatalysts. Silver phosphate, Ag_3_PO_4_, has been reported to possess superior photooxidative capabilities for the degradation of organic dyes under visible light irradiation. However, self-photo corrosion is the main limitation of Ag_3_PO_4_, which restricts its application for only one cycle. Coupling Ag_3_PO_4_ with graphitic carbon nitride (g-C_3_N_4_) is one of the promising techniques to suppress photo-corrosion and effectively enhance photocatalytic performance [5,6,7]. g-C_3_N_4_ is known as a metal-free semiconductor (bandgap energy is 2.7 eV) with a π-conjugated structure and possesses high standard reduction potential (−1.15 eV vs. normal hydrogen electrode (NHE)) to produce more oxygen oxidation species (e.g., hydroxyl radical) [5]. The excellent photocatalytic activity of Ag_3_PO_4_/g-C_3_N_4_ composite is ascribed to the efficient electron–hole separation [8].

Another major problem that restricts photocatalyst application in wastewater treatment is post-treatment difficulty after the degradation process. The powder form photocatalyst undergoes severe agglomeration, and its recovery is challenging, which leads to a high separation operation cost. By immobilizing the photocatalyst into a polymer matrix, such a problem can be overcome. Several polymers have successfully been used as supports for photocatalysts, which includes polyamide, polyvinylidene fluoride (PVDF) [9,10,11], polysulfone (PS) [12,13], polyethersulfone (PES) [14,15], polyurethane (PU) [16,17], and polyacrylonitrile (PAN) [18,19,20].

Lately, PES gains more attention due to its resistance toward UV irradiation and hydroxyl radicals produced in the photocatalytic reaction, thus making it a suitable support material for photocatalysts [21,22,23]. However, most of the studies focused on UV active photocatalyst incorporated into PES matrix as film photocatalyst. To the best of our knowledge, incorporating visible light photocatalyst into PES has not been studied, mainly in the photocatalysis process. Herein, this study focuses on using immobilized Ag_3_PO_4_/g-C_3_N_4_-PES photocatalyst film in the degradation of methyl orange dye. The effects of operational parameters include composite loadings, initial pollutant concentration, initial pH, and film thickness, were investigated.

## 2. Materials and Methods

### 2.1. Materials

Polyethersulfone (PES Ultrason E6020P with M_w_ = 75,000 g/mol) was purchased from Solvay Specialty Polymers, (Jurong Island, Singapore). Thiourea (CH_4_N_2_S) was purchased from HmbG (Kuala Lumpur, Malaysia). N-methyl-2-pyrolidone (NMP) and silver nitrate (AgNO_3_) were purchased from Merck, di-sodium hydrogen phosphate (Na_2_HPO_4_) was purchased from Sigma Aldrich (Petaling Jaya, Malaysia), and methyl orange (MO) was purchased from Bendosen Laboratory Chemicals (Kuala Lumpur, Malaysia). Distilled water was used throughout the study. All chemicals are of analytical grade and were used without any further purification.

### 2.2. Ag_3_PO_4_/g-C_3_N_4_ Photocatalyst Preparation

In a typical synthesis of graphitic carbon nitride (g-C_3_N_4_), 5 g of thiourea was heated in a muffle furnace at 500 °C for 2 h. The resulting yellow powder was washed via sonication in distilled water for 2 h and oven-dried at 60 °C overnight. The Ag_3_PO_4_/g-C_3_N_4_ composite photocatalysts were fabricated by the facile co-precipitation method. Generally, 100 mL of 0.6 M of AgNO_3_ was added into 100 mL of distilled water, containing 0.1 g of dispersed g-C_3_N_4_ powder, and stirred for 1 h. Then, 100 mL of 0.25 M Na_2_HPO_4_ was added dropwise into the suspended solution and stirred for 2 h. The obtained product was collected and washed with distilled water and ethanol before being dried overnight at 60 °C.

### 2.3. PES/Ag_3_PO_4_-g-C_3_N_4_ (P-AgC) Composite Film Preparation

P-AgC was prepared by a previously reported methodology [14]. A certain amount of Ag_3_PO_4_/g-C_3_N_4_ powder was added into the clear PES casting solution, which was prepared by dissolving a fixed amount of PES flakes in the organic solvent (NMP) and stirred at 60 °C for 24 h to uniformly dispersed the Ag_3_PO_4_/g-C_3_N_4_ in the PES solution. The compositions of the prepared film are tabulated in Table 1. The solution mixture was cast on a glass plate (10 cm × 8 cm) using a casting knife with a gap setting of 100 µm. The glass plate was immersed in a nonsolvent media (distilled water) and stored in the dark for a day for the solvent–nonsolvent demixing process and separated the film from the glass. Lastly, the resulting films were air-dried at room temperature for 24 h and ready to be used in photocatalytic studies.

### 2.4. PES/Ag_3_PO_4_-g-C_3_N_4_ (P-AgC) Composite Film Characterization

The crystallinity and phase formation of the PES and PES-Ag_3_PO_4_/g-C_3_N_4_ film photocatalysts were analyzed using an X-ray diffractometer (PHILIPS PW 3040/60, Almelo, Netherlands) with CuKα radiation (λ = 0.154 nm, 30 kV, 30 mA) in the scan range of 2θ = 10° to 80° with a 2°/min of scanning speed. The surface film’s morphology was observed using a scanning electron microscope equipped with an electron dispersive X-ray analyzer (Fei Nova Nanosem 230, Eindhoven, Holland). Meanwhile, the cross-section film morphology was visualized by a scanning electron microscope equipped with an electron dispersive X-ray analyzer (SEM-EDX, HITACHI TM3000, Tokyo, Japan. A 3D measuring laser microscope (OLS5000 OLYMPUS, Hamburg, Germany) was used to capture the film’s surface roughness. The X-ray photoelectron spectra (XPS) of the prepared samples were recorded using a Kratos Analytical Axis Ultra DLD photoelectron spectrometer (Manchester, UK) using an Al Kα radiation monochromatic source. The water contact angle of the film was analyzed by a water surface analysis system (VCA 3000S, Billerica, MA, USA).

### 2.5. Photocatalytic Studies

**Figure 1:**
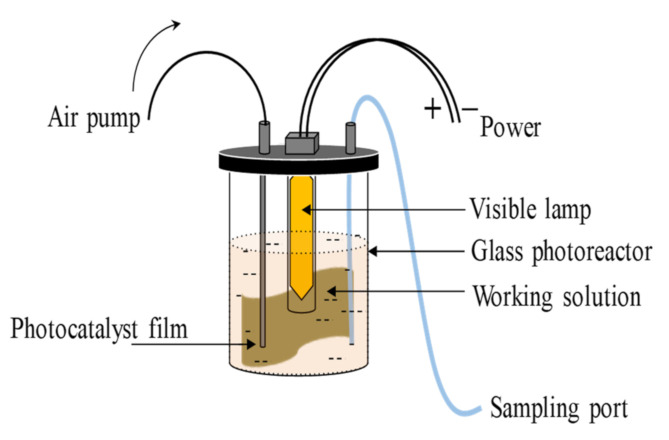
Schematic representation of the photocatalytic reactor setup for photocatalytic activity in a static mode.

The photocatalytic activities of the photocatalyst were evaluated by degradation of methyl orange (MO), a model pollutant, under visible light irradiation The photodegradation was conducted in a 0.5 L glass photoreactor, as shown in Figure 1. Two pieces of P-AgC film photocatalyst (10 cm × 8 cm) were placed around the photoreactor. A known concentration of MO solution (0.3 L) was introduced into the photoreactor. Air was bubbled into the solution to ensure continuous oxygen supply. The film was put under dark condition for 20 min to achieve adsorption–desorption equilibrium before being irradiated by 23 W (fluorescent lamp) for 180 min. Then, 5 mL of sample was withdrawn from the bulk solution at 30 min time intervals, and the residual concentration of the MO was measured using a UV-vis spectrophotometer (Perkin Elmer Lambda 35, Waltham, USA) at λ_max_ = 465.4 nm. The degradation efficiency and the amount of MO degraded were determined using Equations (1) and (2), respectively.

(1) $$\mathrm{Degradation}\;\mathrm{Efficiency}\;(\%)=\frac{C_{o}-C_{t}}{C_{o}}\;\times 100\%$$

(2) $$\mathrm{Amount}\;\mathrm{of}\;\mathrm{MO}\;\mathrm{degraded}\;(\mathrm{mg}/g)=\frac{\left(C_{o}-C_{t}\right)\frac{\mathrm{mg}}{L}\;\times \;\mathrm{volume}\;(L)}{\mathrm{mass}\;\mathrm{of}\;\text{P-AgC}\;\mathrm{film}\;(g)}\;$$

where *C_o_* is the concentration of methyl orange (MO) before irradiation and $C_{t}$ is the concentration of MO at ‘*t*’ time.

Chemical oxygen demand (COD) analysis was performed by digesting 2 mL of MO in a fixed amount of the oxidant reagent (Lovibond) at 150 °C for 1 h by using Thermoreactor RD125 (Lovibond). A colorimeter (DR/890) was used to read the COD concentration. The COD percentage was calculated according to the equation as follows:

(3) $$\mathrm{COD}\;\mathrm{percentage}\;\left(\%\right)=\frac{{\mathrm{COD}}_{o\;}-{\;\mathrm{COD}}_{t}}{{\mathrm{COD}}_{o}}\;\times \;100\%$$

where COD*_o_* and COD*_t_* are the COD concentration initially and at a time “*t*”, respectively.

## 3. Results and Discussion

### 3.1. Characterization of P-AgC Composite Films

**Figure 2:**
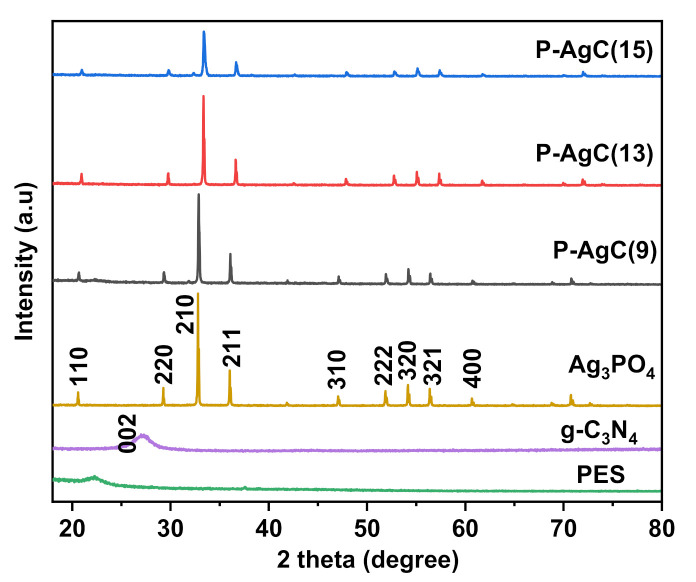
X-ray diffraction patterns of P-AgC in different loadings of Ag_3_PO_4_/g-C_3_N_4_ composite.

The X-ray diffraction patterns of net PES, powder Ag_3_PO_4_, g-C_3_N_4_, and selected P-AgC film photocatalysts are depicted in Figure 2. No peak was observed for bare PES, indicating the amorphous nature of the polymer matrix. The addition of Ag_3_PO_4_/g-C_3_N_4_ into the PES matrix contributed to the appearance of several Ag_3_PO_4_ peaks that was indexed as body-centred cubic phase (JCPDS No. 060505), and no other impurities were observed, indicating that the samples were composed of pure phase Ag_3_PO_4_. No diffraction pattern for g-C_3_N_4_ was observed due to the small amount of usage. It can be concluded that there were no Ag particles present in the composite film.

**Figure 3:**
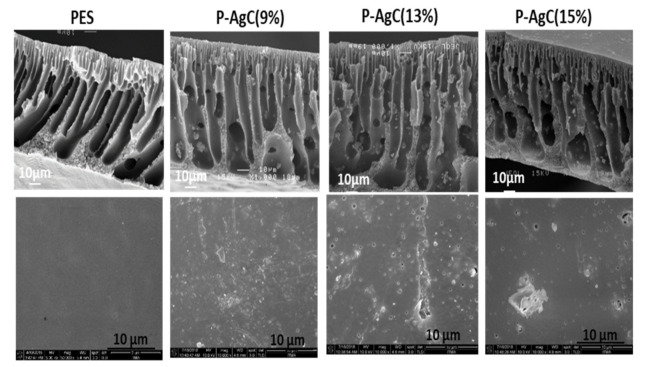
SEM cross-section images and FESEM surface images of PES net, P-AgC (9%), P-AgC (13%), and P-AgC (15%) composite films.

**Figure 4:**
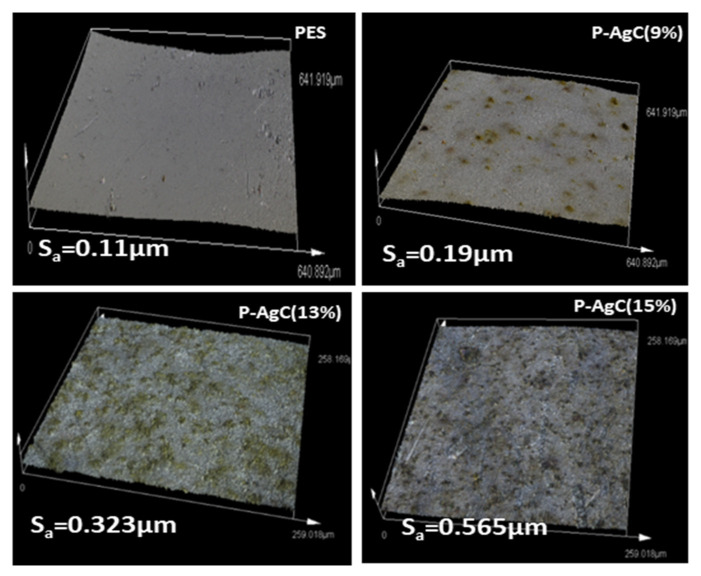
The surface roughness of the neat PES net, P-AgC (9%), P-AgC (13%), and P-AgC (15%) composite films.

**Figure 5:**
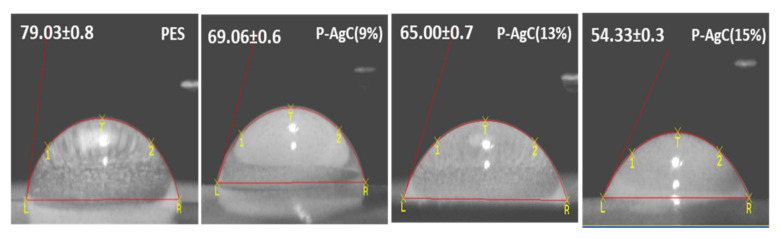
The water contact angle for the neat PES net, P-AgC (9%), P-AgC (13%), and P-AgC (15%) composite films.

The morphology of the PES-Ag_3_PO_4_/g-C_3_N_4_ composite film photocatalyst was observed and depicted in Figure 3. The cross-sectional SEM images well revealed that the PES is made up of a top dense skin layer and finger-like void structures at the bottom layer, which represents asymmetric membranes characteristics. As a result of Ag_3_PO_4_/g-C_3_N_4_ addition, the uniform finger-like microstructures of PES became elongated and non-uniform, with bottle-neck microvoids, appeared at the bottom layer. The presence of the Ag_3_PO_4_/g-C_3_N_4_ composite might have enhanced the casting solution instability and accelerated the demixing process, which facilitated the development of non-uniform microvoids in the composite film [24,25]. The surface morphology and surface roughness of the composite films are evaluated based on FESEM and LSM images. The smooth surface of the neat PES film becomes rougher with the increased Ag_3_PO_4_/g-C_3_N_4_ content. The increase in the average surface roughness may be due to the Ag_3_PO_4_/g-C_3_N_4_ composite aggregation, as shown by the LSM image (Figure 4). The surface hydrophilicity of the films was evaluated by contact angle analysis (Figure 5). By increasing the Ag_3_PO_4_/g-C_3_N_4_ composite content in PES membrane, the water contact angle reduced compared to neat PES, indicating that the hydrophilicity of the composite film had improved. The enhanced hydrophilicity can be due to Ag high affinity toward the water and the existence of hydrophilic groups such as N-H in g-C_3_N_4_ [24,26].

**Figure 6:**
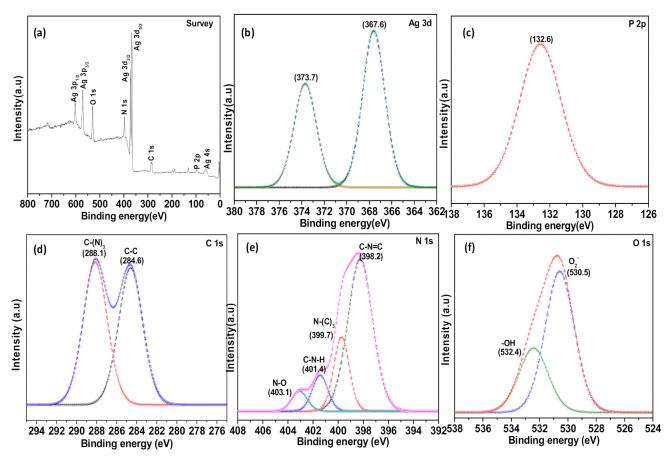
(**a**) Full XPS spectra and high resolution (**b**) Ag 3d peak, (**c**) P 2p peak, (**d**) C 1s peak, (**e**) N 1s peak, and (**f**) O 1s peak of Ag_3_PO_4_/g-C_3_N_4_ composite.

Figure 6 shows the XPS spectra of the as-prepared Ag_3_PO_4_/g-C_3_N_4_ composite, which reveals the chemical compositions and chemical states of the samples. From the XPS survey spectra in Figure 6a, the elements of Ag, P, C, N, and O were detected. Figure 6b shows the high-resolution Ag 3d spectrum and two deconvoluted peaks that ascribe to Ag^+^ in Ag_3_PO_4_ were seen at 373.3 eV and 367.8 eV corresponding to Ag 3d_3/2_ and Ag 3d_5/2_, respectively [5]. The result confirmed that no Ag was formed during the composite preparation. In Figure 6c, the P 2p peak appeared at 132.6 eV, suggesting that the phosphorus in the sample existed in the pentavalent oxidation state (P^5+^) [27]. It can be seen from the C 1s spectrum (Figure 6d) that the curve could be fitted into two peaks located at 284.6 eV, corresponding to the C-C bond with sp^2^ orbital, and the peak located at 288.1 eV could be assigned to CN_3_ bonds in the heterocycle ring of g-C_3_N_4_ [6,28,29]. The N 1_S_ spectrum (Figure 6e) can be deconvoluted into four peaks at 298.2, 399.7, 401.4, and 403.1 eV, which can be attributed to C-N=C, N-(C)_3_, N-H, and N-O in g-C_3_N_4_ structure, respectively. The O 1s peaks (Figure 6f) at 530.5 eV and 532.4 eV are associated with the O_2_^−^ in the Ag_3_PO_4_ and an –OH group or a water molecule on the surface of the composite.

**Figure 7:**
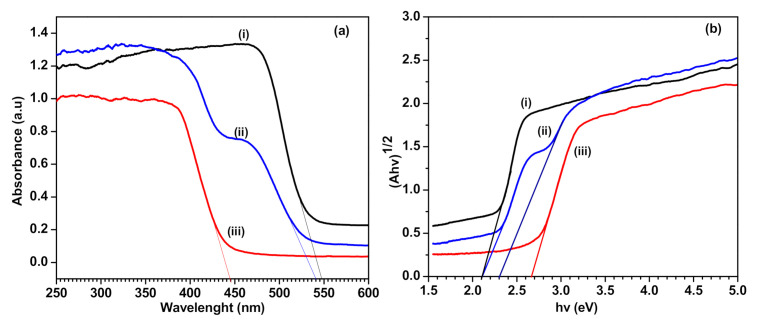
(**a**) UV-vis diffuse reflectant spectroscopy (DRS) spectra and (**b**) band gap energy of (i) Ag_3_PO_4_, (ii) Ag_3_PO_4_/g-C_3_N_4_ composite and (iii) g-C_3_N_4_.

The optical properties of the Ag_3_PO_4_/g-C_3_N_4_ composite photocatalyst were measured using UV-vis diffuse reflectant spectroscopy (DRS). As shown in Figure 7a, the absorption edge of pure Ag_3_PO_4_ and g-C_3_N_4_ were detected at approximately 440 nm and 550 nm, indicating that both the Ag_3_PO_4_ and g-C_3_N_4_ can absorb visible light energy. Meanwhile, the light absorption of the Ag_3_PO_4_/g-C_3_N_4_ composite showed two edges and broad background in the visible light region, which means that the visible light absorption ability was improved. Based on the Kubelka–Munk function, the plots of (Ahv)^1/2^ vs. hv for Ag_3_PO_4_, g-C_3_N_4,_ and Ag_3_PO_4_/g-C_3_N_4_ composite are shown in Figure 7b. The indirect bandgap energies of the Ag_3_PO_4_ and g-C_3_N_4_ were 2.1 eV and 2.5 eV, respectively. For the Ag_3_PO_4_/g-C_3_N_4_ composite, the bandgap for Ag_3_PO_4_ maintained, while the bandgap for g-C_3_N_4_ reduced to 2.3 eV significantly.

### 3.2. The Photocatalytic Activity of the P-AgC Film Photocatalysts

**Figure 8:**
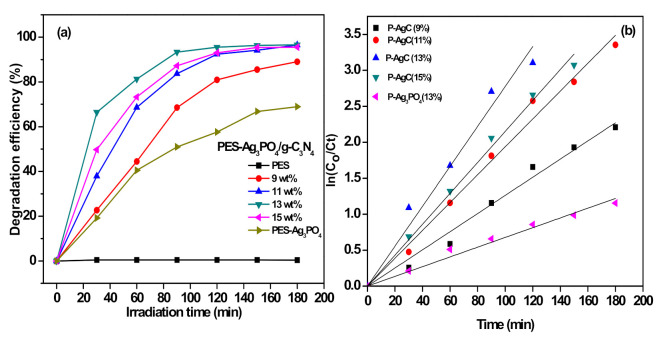
(**a**) Degradation efficiency and (**b**) kinetic study of the degradation of MO by the P-AgC photocatalyst films with different Ag_3_PO_4_/g-C_3_N_4_ content (0–15 wt %) (volume of MO: 0.3 L, number of the film: 2 films, MO concentration: 10 mg/L).

The photocatalytic activity of P-AgC film photocatalysts was evaluated by degrading methyl orange (MO) under visible light irradiation. Before the photocatalytic reaction, the sample was immersed in the MO solution for 30 min in dark to reach the adsorption–desorption equilibrium. Direct photolysis showed no degradation of MO in aqueous solution, which confirmed the stability of MO under visible light irradiation. The degradation efficiency and degradation rate are shown in Figure 8. Net PES did not show any photocatalytic properties. The degradation efficiency of P-AgC (13%) was 97%, which resulted in highest degradation efficiency and the rate of reaction among the samples tested (Table 2). It showed that the presence of carbon nitride enhanced the photocatalytic properties of Ag_3_PO_4_. The enhanced photodegradation efficiency could also be attributed to the increasing hydrophilicity of the photocatalyst film, as evidenced in Figure 5. The improved surface wettability of the film allows more contact between the MO and the Ag_3_PO_4_/g-C_3_N_4_ photocatalyst, leading to a higher MO adsorption on the surface of the photocatalyst film (Figure S1), hence higher photocatalytic efficiency. However, as the Ag_3_PO_4_/g-C_3_N_4_ loadings increased up to 15 wt %, the degradation efficiency decreased. This phenomenon might be due to the agglomeration of Ag_3_PO_4_/g-C_3_N_4_ particles in the PES matrix. The agglomeration resulted in lower active surface area of Ag_3_PO_4_/g-C_3_N_4_ for photon absorption to promote photocatalyst activation. Thus, 13 wt % is the maximum amount of Ag_3_PO_4_/g-C_3_N_4_ that could be immobilized onto the PES film to successfully achieve optimum photocatalytic activity.

**Figure 9:**
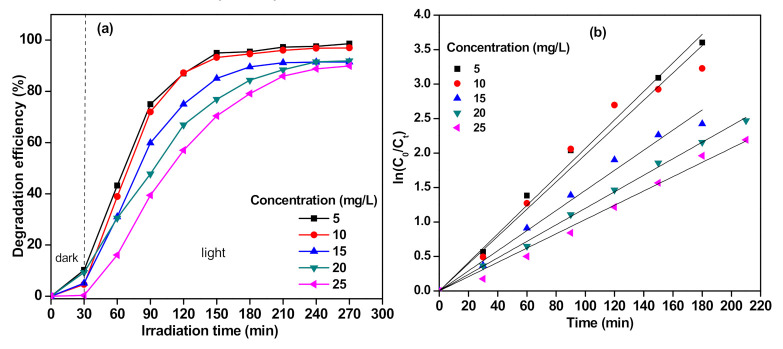
The effect of initial concentration on (**a**) degradation efficiency and (**b**) kinetics of MO degradation in the P-AgC (13%) film photocatalyst (volume of MO: 0.3 L, number of films: 2 films).

The effect of MO initial concentration toward degradation efficiency is shown in Figure 9, and details are outlined in Table 3. It was found that the degradation efficiency decreased as the MO concentration increased due to the screening effect of MO dye that reduced the path length of the photon that arrived on the surface of the photocatalyst. At low MO concentration, the number of reactive radicals (•OH and •O_2_^−^) is more than the number of MO molecules, increasing the chance of collision between the pollutant and the active site of catalyst. Hence, it increased the tendency of adsorption and oxidation of MO on the surface of catalyst [30]. As the MO concentration increased, the adsorption of substrate molecules on the photocatalyst surface suppressed the generation of reactive radicals. Practically, the reaction cannot be enhanced as the amount of active sites and the concentrations of the hydroxyl radicals are constant [31].

**Figure 10:**
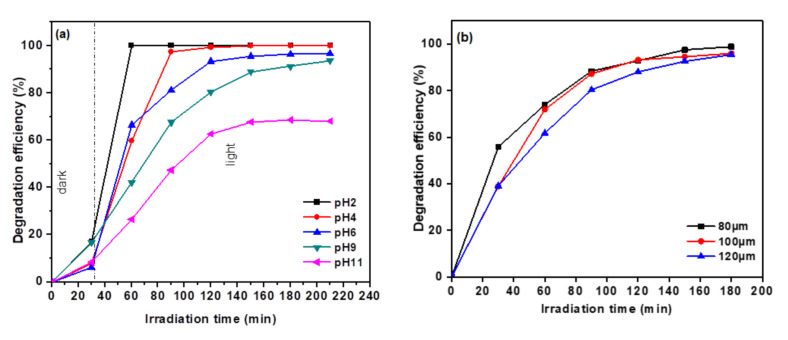
(**a**). Effect of initial pH of MO solution and (**b**) film thickness on degradation efficiency of P-AgC (13%) (volume of MO: 0.3 L, number of the film: 2 films, MO concentration: 10 mg/L).

The effect of pH in the degradation efficiency of MO was studied by varying the pH of MO solution from 2 to 11 using 1 M of hydrochloric acid (HCl) and sodium hydroxide (NaOH), and the results are depicted in Figure 10a. It can be seen that the photodegradation favored acidic conditions as the degradation efficiency achieved 100% within 30 min under irradiation at pH 2. In acidic conditions, Ag_3_PO_4_ particles dissolved in the solution to form Ag^+^ ions. The presence of Cl^−^ anion leads to the formation of AgCl. AgCl is known to possess excellent photocatalytic properties; hence, this AgCl formation hastens the rate of MO degradation [32,33,34]. However, as the initial pH increased up to 11, the degradation efficiency was depleted. The competitive adsorption between OH^−^ and anionic MO onto the catalyst surface led to the reduction in degradation efficiency of MO in alkaline condition. The pH_pzc_ of Ag_3_PO_4_ is 6.8, which made its surface charge negative under basic condition [35]. Thus, strong repulsion existed between the anionic MO and negatively charged Ag_3_PO_4_ surface, resulting in poor degradation activity.

The effect of film thickness on the MO degradation is illustrated in Figure 10b. As the film thickness increased, the light penetration of the top layer of the film decreases, leading to a reduction in the activation of the Ag_3_PO_4_/g-C_3_N_4_ catalyst in the membrane matrix; hence, it reduces the degradation efficiency of the composite film photocatalyst.

**Figure 11:**
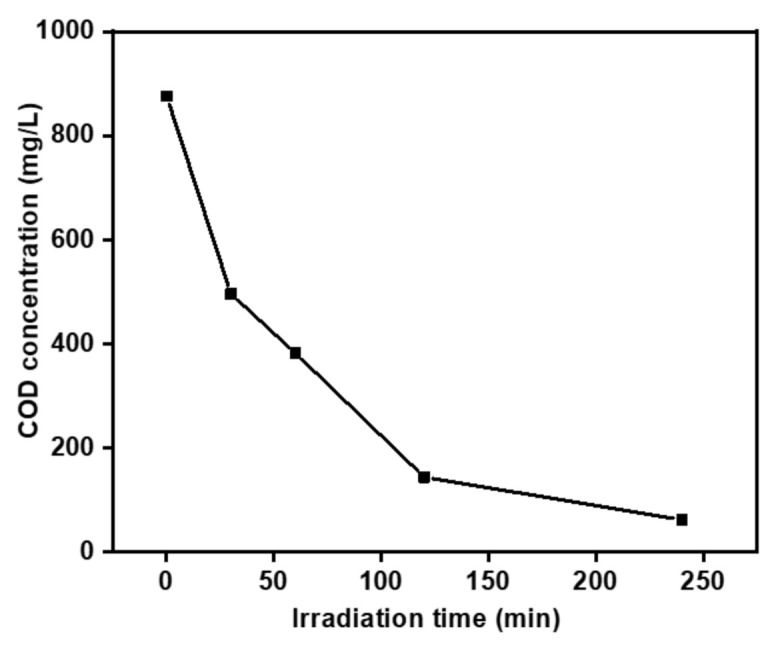
Chemical oxygen demand (COD) removal of P-AgC (13).

To ensure that the MO removal was via the photodegradation process, COD analysis was conducted. As depicted in Figure 11, the COD concentration decreased with increasing reaction time, confirming the degradation of the MO.

### 3.3. Reusability of P-AgC (13%)

**Figure 12:**
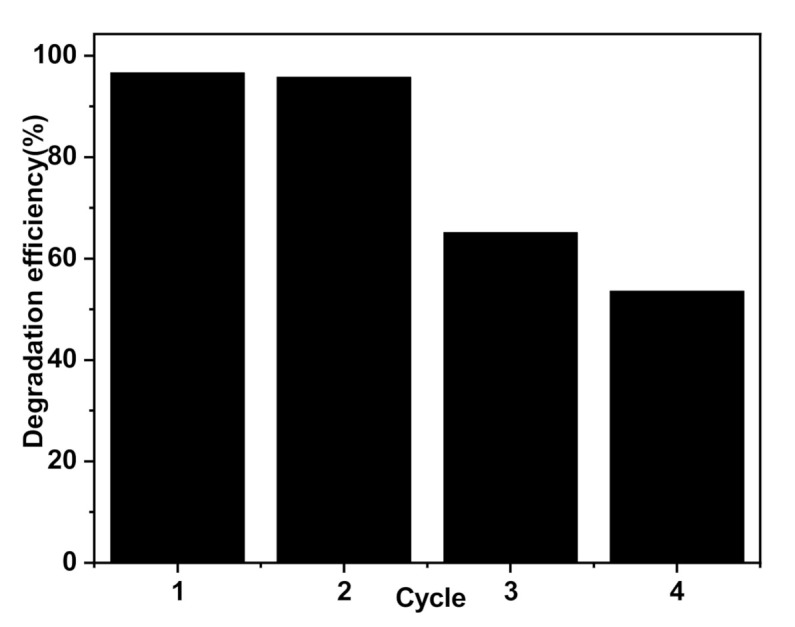
Reusability study of P-AgC 13%) (volume of MO: 0.3 L, number of the film: 2 films, MO concentration: 10 mg/L).

For the reusability study, the photodegradation of MO (10 mg/L) by P-AgC (13%) film was tested at pH 6.2 for 3 h of irradiation. The catalyst was washed with deionized water and dried before being used in new MO solution for every cycle. After recycling four times, as shown in Figure 12, the degradation efficiency decreased sharply from 96.6% to 53.5%, which indicates that the P-AgC (13%) film possesses excellent photocatalytic stability up to three cycles. The high-resolution XPS spectrum of Ag 3d (Figure S2) of the film after six experimental cycles showed dominant peaks of Ag^0^ at binding energy 368.2 eV and 374.2 eV in P-AgC (13) in addition to the peaks attributed to the Ag^+^ at 367.8 eV and 373.6 eV. Hence, the decreased in the activity may be due to the formation of Ag particles in the film.

### 3.4. Mechanism of Photodegradation of MO and Effect of Scavengers

**Figure 13:**
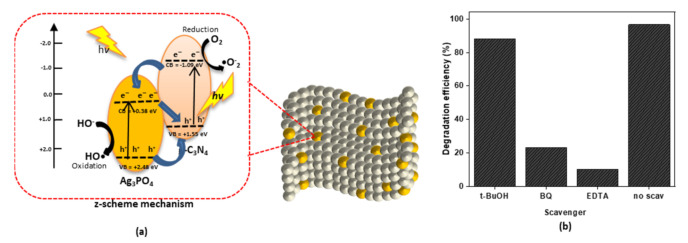
(**a**) Plausible mechanism of photodegradation of MO in the P-AgC (13%) composite film photocatalyst and (**b**) effect of radical scavengers (*tert*-butanol, *p*-benzaquinone, sodium EDTA) toward degradation efficiency of MO.

The mechanism for photodegradation of MO was postulated based on the activation of Ag_3_PO_4_/g-C_3_N_4_ composite photocatalyst by low energy visible light photons to generate electron–hole pairs (Figure 13a). Through a series of reactions between electrons and adsorbed oxygen and between holes and adsorbed water, OH radicals formed to eventually degrade MO molecules. The superior photocatalytic activity of P-AgC is mainly due to the performance of both Ag_3_PO_4_ and g-C_3_N_4_ to form electron–hole pairs for reactive species production in the solution.

The conduction band (E_CB_) and valence band (E_VB_) potentials of the photocatalyst were calculated using Equations (4) and (5), respectively.

(4) $$E_{\mathrm{CB}\;}=\chi \;-{\;E}^{e}\;-\;0.5E_{g}$$

(5) $$E_{\mathrm{VB}}=E_{\mathrm{CB}}{+E}_{g}$$

where E^e^ is the energy of free electrons on the hydrogen scale (4.5 eV); E_g_ is the bandgap energy, and χ is the electronegativity of semiconductor, which was calculated using the following equation:

(6) $$\chi =[x{\left(A\right)}^{a}x{\left(B\right)}^{b}x{\left(C\right)}^{c}{]}^{1/(a+b+c)}$$

where a, b, and c are the number of atoms in the compounds.

Based on the E_g_ and χ values of 2.1 eV and 5.93 eV, the calculated E_CB_ and E_VB_ for Ag_3_PO_4_ versus normal hydrogen electrode (NHE) are +0.38 eV and +2.48 eV, respectively [36,37]. Meanwhile, for the g-C_3_N_4_, the E_g_ value is 2.65 eV and the χ values is 4.73 eV; hence, the estimated E_CB_ and E_VB_ were −1.09 eV and +1.55 eV, respectively, which agreed with previous research [38,39,40].

When the visible light irradiated the Ag_3_PO_4_/g-C_3_N_4_ composites, the photons would be absorbed by Ag_3_PO_4_ and g-C_3_N_4_, generating electron–hole pairs. The photogenerated electrons on the conduction band (CB) of g-C_3_N_4_ react with adsorbed O_2_ to form •O_2_^−^ radicals, because CB of g-C_3_N_4_ is more negative than the potential of O_2_/•O_2_^−^(E^o^(O_2_/•O_2_^−^) = −0.33 eV/NHE) [41]. Since the CB edge potential of Ag_3_PO_4_ (+0.38 eV) is more positive than the standard redox potential of O_2_/•O_2_^−^, Ag_3_PO_4_ cannot reduce O_2_ to •O_2_^−^. However, the electrons in the CB of Ag_3_PO_4_ can be transferred to adsorbed oxygen molecules to produce H_2_O_2_, because the CB of Ag_3_PO_4_ is more negative than E^o^ (O_2_/H_2_O_2_ = +0.682 eV vs. NHE). The produced H_2_O_2_ molecules react with electrons to produce •OH radicals [37]. Through the z-scheme mechanism, the electrons can also be transferred to the valence band (VB) of the g-C_3_N_4_ [42]. On the other hand, the holes in the valence band of Ag_3_PO_4_ can oxidize the pollutant directly, since the VB potential of Ag_3_PO_4_ is more positive than the E^o^ (OH^−^/•OH = +2.4 eV).

To study the main reactive species involved in the photodegradation of MO, tert-butanol, p-benzoquinone, and sodium EDTA were used as a trapper for hydroxyl radicals (•OH), superoxide anion radicals (•O_2_^−^), and holes (h^+^), respectively, in the reaction system, and the results are shown in Figure 13b. When p-benzoquinone and sodium EDTA were used, the degradation efficiency declined to 23% and 10%, respectively. It can be concluded that •O_2_^−^ and h^+^ were the dominant radical species involved in the degradation of MO.

## 4. Conclusions

A novel PES-Ag_3_PO_4_/g-C_3_N_4_ was successfully fabricated by the phase inversion technique. The incorporation of Ag_3_PO_4_/g-C_3_N_4_ into the PES matrix widened the finger–like microvoid structure and formed a bottle-neck structure. The film hydrophilicity, surface roughness, and porosity were also enhanced significantly. The dye degradation experiment showed that 13 (wt %) is the optimum amount of the Ag_3_PO_4_/g-C_3_N_4_ composite to be loaded into the PES matrix, which resulted in the highest photocatalytic activity. The PES-Ag_3_PO_4_/g-C_3_N_4_ could be used up to three cycles before the performance was reduced to half. The •O_2_^−^ and h^+^ were the dominant species in the degradation of MO.

## Figures and Tables

**Table 1:** The casting solution compositions for the preparation of P-Ag_3_PO_4_/g-C_3_N_4_ film photocatalyst.

PES (wt %)	Ag_3_PO_4_/g-C_3_N_4_ (wt %)	NMP (wt %)	Label
15	0	85	PES
15	9	76	P-AgC (9%)
15	11	74	P-AgC (11%)
15	13	72	P-AgC (13%)
15	15	70	P-AgC (15%)

**Table 2:** Kinetic data and amount of MO degraded by the P-AgC photocatalyst films.

Sample	Degradation Efficiency (%)	k_obs_ (min^−1^)	Rate (mg/Lmin)	Correlation Factor (R^2^)
P-Ag_3_PO_4_ (13%)	68.69	0.007	0.067	0.994
P-AgC (9%)	89.01	0.013	0.126	0.994
P-AgC (11%)	96.50	0.020	0.193	0.996
P-AgC (13%)	96.58	0.028	0.277	0.991
P-AgC (15%)	95.50	0.022	0.215	0.998

**Table 3:** Kinetic data and amount of MO degraded at different initial concentrations using P-AgC.

MO Concentration (mg/L)	Degradation Efficiency (%)	k_obs_ (min^−1^)	Rate (mg/Lmin)	Amount Degraded (mg/g)	Correlation Factor (R^2^)
5	98.61	0.0207	0.103	3.101	0.997
10	96.95	0.0198	0.198	5.952	0.989
15	91.45	0.0146	0.219	7.435	0.995
20	91.89	0.0119	0.240	11.675	0.999
25	89.93	0.0104	0.259	14.336	0.996

## Data Availability

The data presented in this study are available on request from the corresponding author.

## Supplementary Materials

The following are available online at https://www.mdpi.com/article/10.3390/polym13111746/s1.
