# Supplementary material for: PES-Ag3PO4/g-C3N4 Mixed Matrix Film Photocatalyst for Degradation of Methyl Orange Dye
Source: Polymers (Basel). 2021 May 27;13(11):1746. doi: 10.3390/polym13111746 (PMC8198369; doi:10.3390/polym13111746)
Supplement: Supplementary file 1 [file polymers-13-01746-s001.zip › polymers-1145147-supplementary.pdf]

polymers

MDPI

Supporting Information

PES-Ag_3PO_4/g-C_3N_4 Mixed Matrix Film Photocatalyst for Degradation of Methyl Orange Dye

Hayati Mohamad Mukhair [1], Abdul Halim Abdullah [1,2,*], Zulkarnain Zainal [1,2] and Lim Hong Ngee [1,2]

The adsorption studies for PES and P-AgC with different Ag_3PO_4/g-C_3N_4 loading were conducted under dark condition for 30 min. The percentage of adsorption was illustrated in Figure S1.

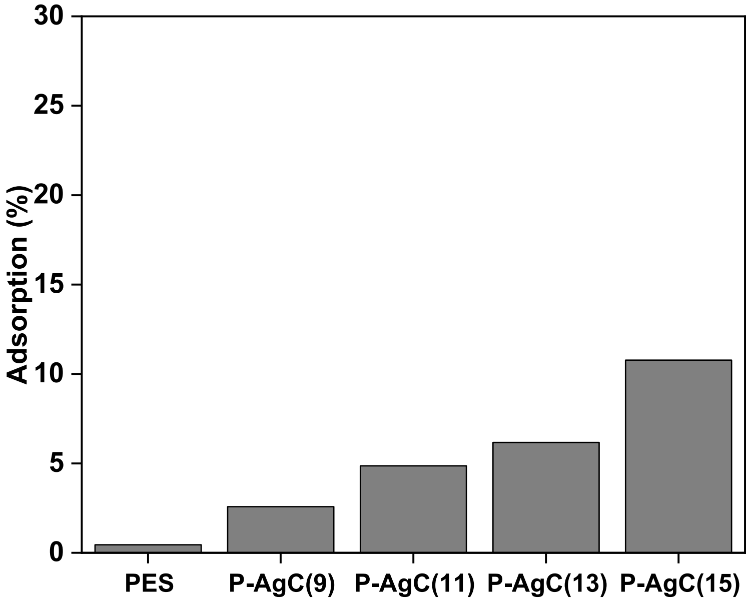

Figure S1. Percentage of MO adsorption by PES, P-AgC(9), P-AgC(11), P-AgC(13), and P-AgC(15).

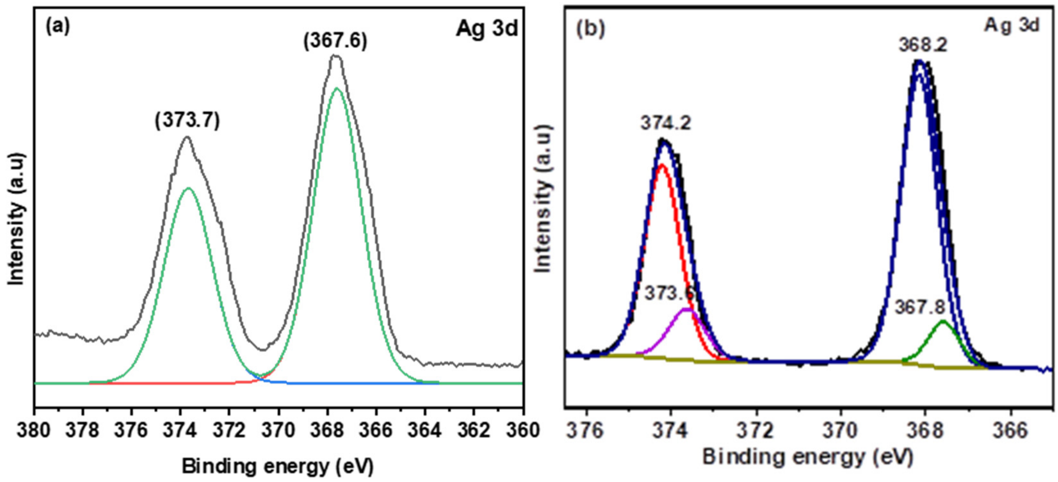

Figure S2. Binding energy of high resolution Ag 3d spectra of (**a**) P-AgC(13) before reaction and (**b**) P-AgC(13) after sixth cycles.
